# Analysis of CD20 and PD-L1 levels on small extracellular vesicles (sEV) produced by DLBCL cells and EBV-transformed B cells, and potential role in T cell inhibition

**DOI:** 10.1186/s40164-024-00518-2

**Published:** 2024-05-17

**Authors:** Hussein Akil, Hafidha Bentayeb, Marine Aitamer, Chantal Vignoles, Julie Abraham, Nathalie Gachard, Agnès Olivrie, Anne Guyot, Jessica Gobbo, Jean Feuillard, Hamasseh Shirvani, Danielle Troutaud

**Affiliations:** 1 CRIBL, UMR CNRS 7276 -INSERM U1262, CBRS, Université de Limoges, 2 Rue du Docteur Marcland, 87025 Limoges Cedex, France; 2 CAPTuR UMR INSERM U1308, Facultés de Médecine et de Pharmacie, Université de Limoges, 2 Rue du Docteur Marcland, 87025 Limoges Cedex, France; 3 Service d’Hématologie Clinique, CHU de Limoges, 2 Avenue Martin Luther King, 87000 Limoges, France; 4 Laboratoire d’hématologie, CHU de Limoges, 2 Avenue Martin Luther King, 87000 Limoges, France; 5 Service d’Anatomie Pathologique, CHU de Limoges, 2 Avenue Martin Luther King, 87000 Limoges, France; 6 INSERM 1231, Label Ligue National Contre le Cancer and Label d’excellence LipSTIC of Dijon; Early Phase Unit INCa CLIP, Anti-Cancer Center Georges-François Leclerc, Dijon, France; 7 Institut Roche, 30, Cours de L’île Seguin, 92650 Boulogne-Billancourt, France

**Keywords:** DLBCL, Human lymphoblastoid cell lines, sEV, Exosomes, CD20, PD-L1, T cells, Immunosuppression

## Abstract

Increasing evidence supports a role for small extracellular vesicles (sEV, including exosomes) in Diffuse Large B-cell lymphoma (DLBCL) progression and resistance to treatment. CD20 and PD-L1 are found on DLBCL-derived sEV, but little is known about their patient-level heterogeneity. Moreover, the capacity of PD-L1^+^ sEV to modulate T cells needs to be clarified. Herein we analyzed sEV produced by human DLBCL cell lines and EBV-transformed B cell-lymphoblastoid cell lines (LCLs), a model allowing autologous T cell co-cultures. We determined CD20 and PD-L1 levels on plasma sEV from patient samples vs healthy volunteers (HV). sEV functional relevance was also investigated on CD4^+^ and CD8^+^ T cells. sEV derived from all cell lines showed an enrichment of CD20 and a high glycosylated PD-L1 expression when compared to cell lysates. High PD-L1 expression on LCL-derived sEV was associated with higher CD4^+^ and CD8^+^ T cell apoptosis. In patients, plasma sEV concentration was higher vs HV. Compared to sEV-CD20 level that seemed higher in patients, PD-L1 level in sEV was not different from those of HV. A high glycosylated PD-L1 level was shown in sEV from both patients and HV plasma samples, that was associated with the same inhibiting effect on activated T cells. We conclude that sEV derived from EBV-transformed B cells realize an immunosuppressive role that involved cell–cell interaction and probably at least PD-L1. Furthermore, our findings suggest the potential of circulating sEV as a source of biomarkers in DLBCL, notably to have information on immunotherapeutic target levels of parental tumor cells.

**Supplementary Information:**

The online version contains supplementary material available at 10.1186/s40164-024-00518-2.

**To the editor,**

In Diffuse large B-cell lymphomas (DLBCL), even though PD-L1 expression has been associated with poor overall survival [1], most patients with relapsed/refractory DLBCL are less sensitive to PD-1 blockade [2]. Few subtypes of NHLs with specific genetic alterations or immunologic properties appear to be more responding, including Epstein-Bar virus (EBV)-associated lymphomas, where PD-L1 expression is upregulated through EBV infection [3]. However, PD-L1 expression is not restricted to tumor cell surface. In solid tumors PD-L1 was also found on extracellular vesicles such as exosomes that could limit the clinical benefit of PD-1/PD-L1 immunotherapy [4, 5]. Exosomes are small extracellular vesicles (sEV, 50–150 nm) of endosomal origin, secreted by normal and tumoral cells during exocytic fusion of multivesicular bodies (MVBs) with the plasma membrane [6]. They regulate intercellular communication by transfer of signaling molecules like proteins, lipids and nucleic acid cargos. Current knowledge shows their important role in the development and progression of cancer, including DLBCL [7, 8]. Interestingly, they may also contribute to drug resistance. We have previously analyzed CD20 levels on sEV derived from ABC and GCB DLBCL cell lines, and demonstrated in a preclinical model sEV role in protecting tumors from the rituximab cytotoxicity [9]. However, heterogeneity of CD20 level on circulating sEV has not been evaluated in patients. Of note, enhanced PD-L1^+^-sEV in the plasma of DLBCL patients compared to healthy volunteers (HV) was recently suggested [10]. In this work, we first characterized CD20 and PD-L1 expression on sEV produced by EBV-transformed B cell-lymphoblastoid cell lines (LCLs), known to express high levels of PD-L1 [11], compared to those of DLBCL cell lines. We used sEV from LCLs to explore the functional interaction of sEV on autologous T cells. Finally, we investigated CD20 and PD-L1 levels on plasma sEV samples derived from DLBCL patients vs HV, and analyzed sEV functional relevance on CD4^+^ and CD8^+^ peripheral T cells.

sEV production (included exosomes) by all cell lines was characterized for size distribution and concentration using nanoparticle tracking analysis (NTA), and protein markers (i.e. Alix, TSG101, CD81 and CD63) were confirmed by western blot analysis of sEV lysates (Fig. 1A, B). CD20 and PD-L1 expressions were examined at the cellular and vesicular levels by western blot and flow cytometry. CD20 level found in LCL- and DLBCL-derived sEV was higher than those of parental cell lines, suggesting an enrichment of CD20 in sEV (Fig. 1B). Interestingly, lower expression of CD20 by LCL-derived sEV was associated, as for DLBCL-derived sEV, with lower binding capacity to rituximab (i.e. C1504 LCL, Fig. S1) and CD20 targets on DLBCL derived-sEV act also as “decoy-receptors” for obinutzumab (GA101) (Fig. S2). High expression of PD-L1 was observed in all LCL-derived sEV that was relatively homogenous, in contrast to those of DLBCL. PD-L1 level on sEV was also analyzed by flow cytometry after immunocapture using anti-CD81 coated magnetic beads (Fig. 1D, E). As we previously reported for CD20 [9], PD-L1 level was not related to the DLBCL subtype, but seemed reflect that of parental cells (Fig. 1B, C, E). Of note, colocalization of PD-L1 and CD63 in MVBs was observed using confocal microscopy suggesting PD-L1 in the precursor form of exosomes (Fig. 1F). Interestingly, using E1L3N anti-PD-L1 antibody that recognizes glycosylated PD-L1, we observed higher glycosylated PD-L1 levels in sEV lysates derived from LCL, and with a lesser extend from DLBCL, compared to cell lysates (Fig. 1B). As N-linked glycosylation of PD-L1 was shown to increase PD-L1/PD-1 interaction, and consequently immunosuppression [12], results strongly suggest a high immunomodulatory capacity of sEV, notably with those from LCL.

**Fig. 1:**
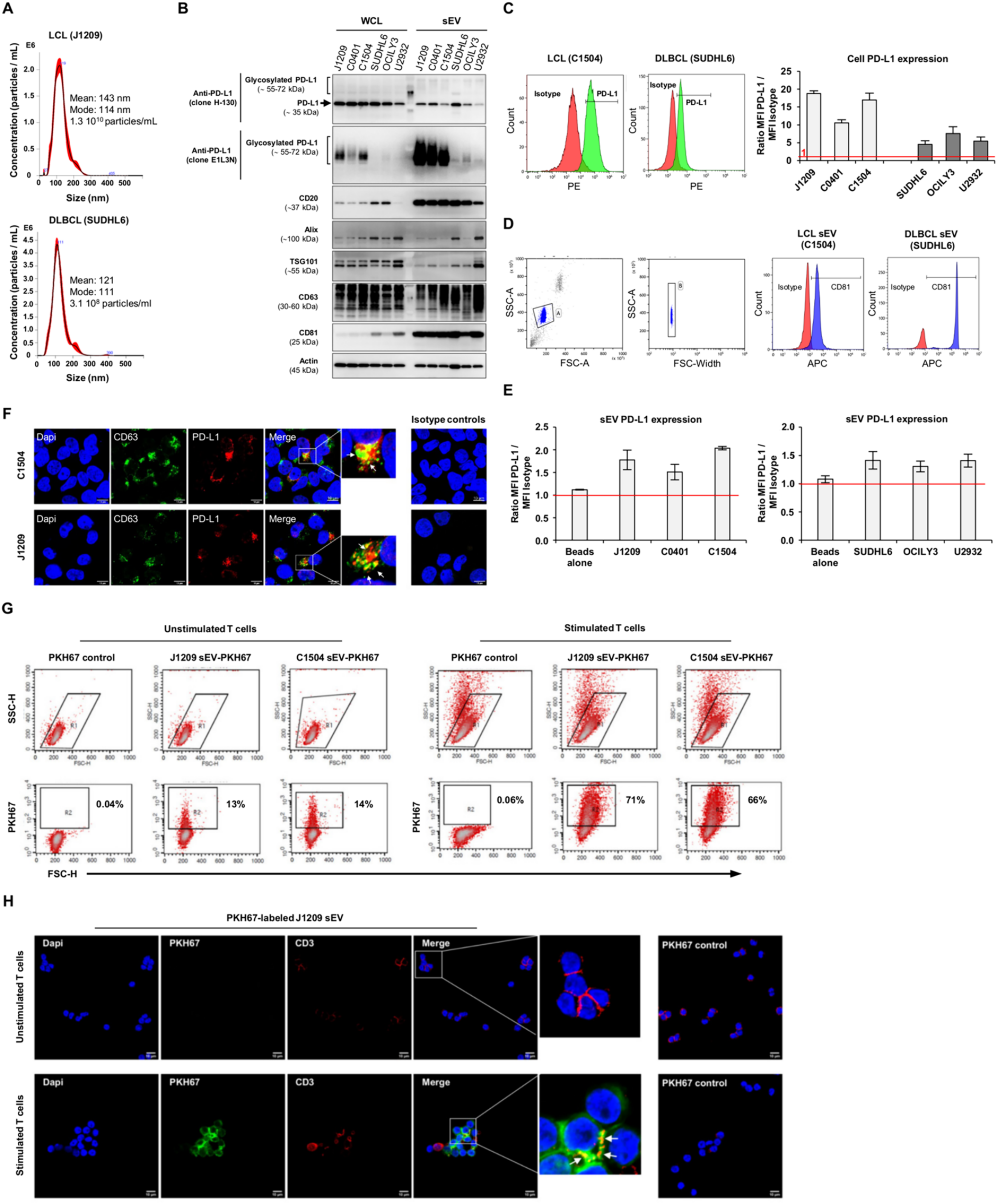
Characterization, phenotypic and functional analysis of small extracellular vesicles (sEV) produced by human lymphoblastoid cell lines (LCLs) as compared to DLBCL cell lines. **A** Nanoparticles tracking analysis and representative plots showing size distributions and concentrations of EV produced by 20.10^6^ LCL (i.e. J1209) and 30.10^6^ DLBCL (i.e. SUDHL6) cell lines after 72 h of culture. Plots represent the mean value (black line) with SD (red shaded area) from 5 recordings. **B** Western blot analysis of PD-L1 (total and glycosylated form) and CD20 protein expression in whole cell lysates (WCL) and sEV lysates for LCL (J1209, C0401, C1504), GCB (SUDHL6) and ABC (OCILY3, U2932) cell lines. 10 µg of proteins extracted from WCL and sEV were loaded. CD81 and CD63 were used as sEV-protein markers, and TSG101 and Alix as specific exosomes markers. Actin was used as loading protein control. PD-L1 glycosylation was confirmed after western blot analysis in the presence or absence of a recombinant glycosidase, PNGase F (data not shown). **C** Flow cytometry analysis of surface PD-L1 expression on LCLs (J1209, C0401 and C1504) and DLBCL (SUDHL6, OCILY3 and U2932) cell lines. Representative histograms of are shown (left) for C1504 and SUDHL6. Results (right) represent the means ± SD of the Mean Fluorescence Intensity (MFI) ratio (MFI PD-L1/MFI isotype control) from 3 independent experiments. **D** Representative flow cytometry gating strategy used to identify anti-CD81 immunocaptured sEV (left). Representative histograms of CD81 staining (anti-CD81-APC or isotype control conjugated antibody) on beads-sEV complexes are shown for C1504 and SUDHL6 (right) showing that the majority of sEV samples were CD81 positive (> 93%) which confirms the effectiveness of sEV immunocapture. **E** Flow cytometry analysis of PD-L1 expression on sEV for 3 LCL and 3 DLBCL cell lines. Data are expressed as means ± SD of MFI ratio of PD-L1 staining to isotype control, from 2 (LCL) and 3 (DLBCL) independent experiments. **F** Representative confocal microscopy analysis images of PD-L1 (red) and CD63 (green) localization are shown in J1209 and C1504 permeabilized LCLs. Co-localization of CD63 and PD-L1 staining are indicated in the merged images and in the zoomed insets (white arrows). Nuclei were counter stained with DAPI. Isotype controls staining are shown in the right. **G** Flow cytometry analysis of the interaction (2 h) of PKH67-labeled sEV derived from J1209 and C1504 LCLs with stimulated and unstimulated primary allogenic T cells. Representative contour plots showing primary T cells gating based on scatters parameters (top) and PKH67 fluorescence vs forward scatter channel (FSC) (bottom) are shown. Data are representative of 2 independent experiments realized in duplicate. **H** sEV uptake by human primary T cells was also analyzed by confocal microscopy from the same cultures. Representative analysis showing uptake of PKH67 labeled sEV (green) derived from J1209 LCL by stimulated and unstimulated primary T cells (stained with anti-CD3-APC, red) after 2 h of incubation. Co-localization of CD3 and PKH67 staining are indicated in the merged images and in the zoomed insets (white arrows). Nuclei was stained with DAPI. PKH67 controls are shown in the right. Data are representative of 2 independent experiments.

We thus explored the immunomodulatory capacity of LCL-derived sEV using PKH-labeled sEV. We showed that LCLs-derived sEV are captured by peripheral T cells that was strongly enhanced after activation (Fig. 1G, H). Surprisingly, using autologous T lymphocytes, LCL-derived sEV induced apoptosis in CD4^+^ and CD8^+^ after 24 h and 48 h, as demonstrated for sEV with high PD-L1 (i.e. derived from J1209 LCL) (Fig. 2A–C). Of note, apoptosis was reduced with sEV carrying lesser PD-L1 levels (i.e. derived from C0401 LCL, data not shown). Upregulation of death receptors after T cell activation could be also involved in this apoptosis as previously reported with DLBCL cell line-derived exosomes and a human T cell line [7].

**Fig. 2:**
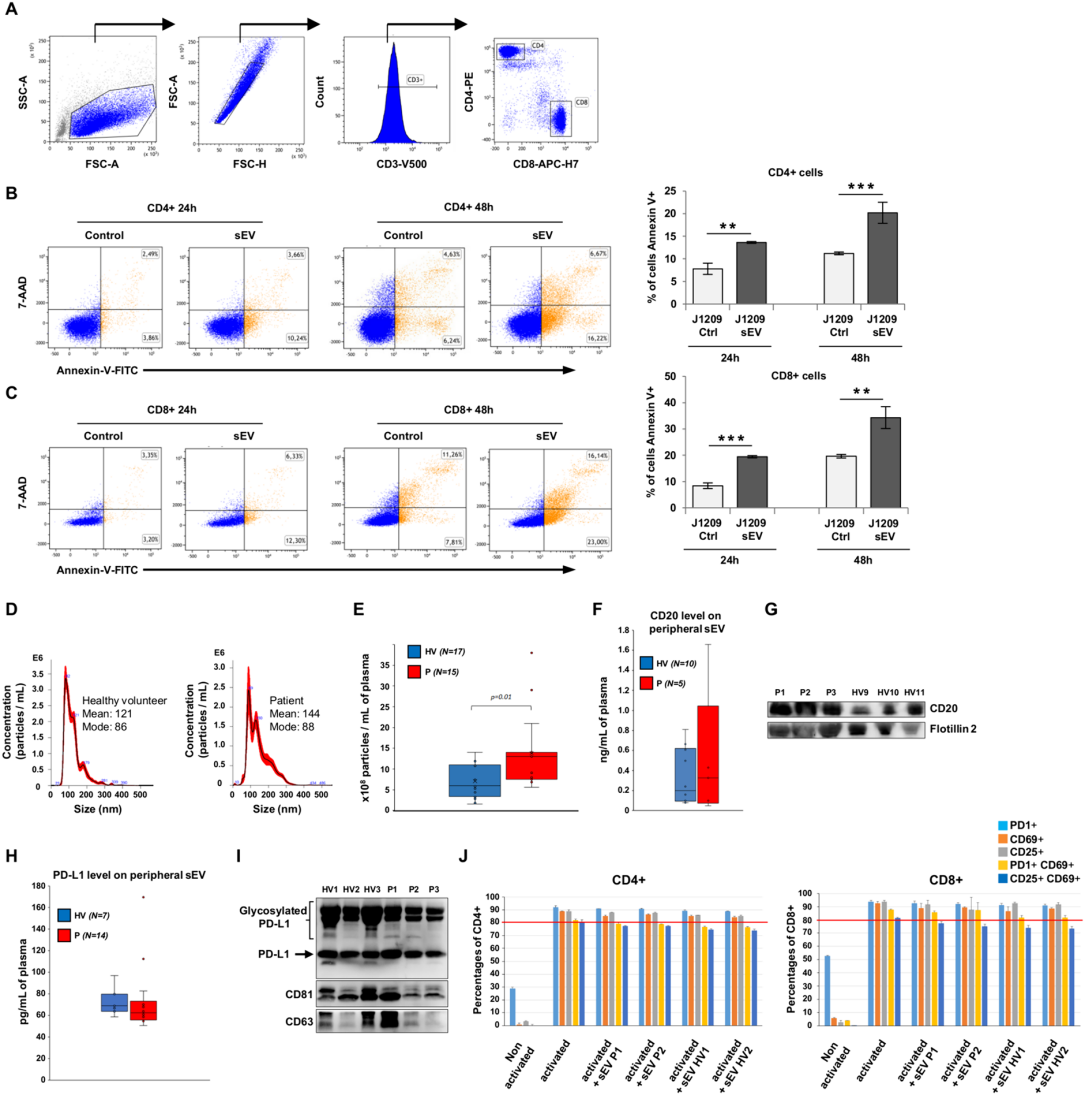
Evaluation of the immunomodulatory function of LCL-derived sEV in an autologous model, and of plasma sEV derived from patients. **A** Representative contour plots showing the gating strategy used to identify purified autologous CD4^+^ and CD8^+^ peripheral T cells. sEV from LCL with a high PD-L1 level (i.e. J1209, 5 µg) were co-incubated (sEV) or not (control) with autologous activated T cells for 24 and 48 h. Representative cytograms of activated CD4^+^ (**B**) and CD8^+^ (**C**) T cells examined for apoptosis following AnnexinV-FITC/7-AAD staining. Percentages of Annexin V^+^ CD4^+^ and Annexin V^+^ CD8^+^ T cells are shown (right). Data are the means ± SD from 3 independent experiments. **, ***: p < 0.01 or 0.001 respectively. **D** Characterization of plasma derived-sEV from newly diagnosed DLBCL patients compared to age-matched healthy donors. Representative NTA of sEV obtained from healthy volunteers (HV, N = 17) and patients (P, N = 15) plasma samples. Plots represent the mean value (black line) with standard error (red shaded area) of 4 recordings. **E** Plasma derived-sEV from DLBCL patients revealed higher concentrations than those of HV. Box plots indicating median and quartiles are done for the two groups, and means are indicated by crosses. sEV were purified from HV and DLBCL (P, patients) plasma samples and analyzed by **F** ELISA and **G** western blotting for the expression of CD20. For ELISA results are expressed as box plots indicating median and quartiles for the two groups (N = 10 among 18 samples tested and N = 5 among 16 samples tested for HV and DLBCL patients respectively, remaining samples were apparently negative as they were below the detection threshold). Flotillin 2 was used as sEV marker protein. Quantification of PD-L1 levels were also performed by ELISA (**H**) with data expressed as box plots indicating median and quartiles for the two groups and western blot (**I**) where CD81 and CD63 expressions were used as sEV marker proteins for western blot analysis. **J** Functional activity of plasma-derived sEV from 2 patients (sEV P1 or P2) or from 2 healthy volunteers (sEV HV1 or HV2) on allogenic T cells activated or not for 48 h. Markers commonly related to T cell activation (i.e. CD25, CD69) and PD1 expression were analyzed by flow cytometry on CD4^+^ and CD8^+^ T cells. Histograms represent the percentage of positive T cells ± SD for each activation marker, alone or in association (double positive PD1^+^CD69^+^ and CD25^+^CD69^+^ T cells) as compared to respective isotype Ab staining (triplicate). The horizontal line indicates % of CD25^+^CD69^+^ positive cells in activated T cell cultures without sEV. The gating strategy used for the identification of CD4^+^ and CD8^+^ peripheral T cells from PBMCs is shown in Fig. S3

To address the role of PD-L1^+^-sEV in patients, we characterized sEV isolated from plasma samples of 15 DLBCL patients versus 17 HV using the NTA technology. No difference in particle size was found; however, sEV concentration was significantly higher in plasma of DLBCL patients than in HV samples (Fig. 2D, E). As for DLBCL cell lines, plasma sEV concentration seemed not influenced by the DLBCL subtype (data not shown). Interestingly, CD20 and PDL-1 strong expressions were demonstrated in sEV lysates of patients. CD20 levels tended to be increased in sEV from patients compared to HV (ELISA and western blot analysis, Fig. 2F, G respectively). We observed a great variability of CD20 level on patient sEV samples using ELISA that could reflect that of tumor cells, while this information was not available with the immunohistochemical analysis of parental tumor biopsy samples (data not shown). Indeed, due to data limitations, notably semi-quantification of CD20 expression on tumor tissue that was not performed, we cannot conclude on a possible correlation between CD20 expression on tumor samples and sEVs. Such a correlation would make it possible to propose monitoring the CD20 level on plasma sEV of patients as a more sensitive method to inform clinicians on the CD20 status of parental tumor cells. sEV PD-L1 expression was more homogeneous than for CD20, and PD-L1 level was high in both HV and patient samples (ELISA and western blot analysis, Fig. 2H and I respectively); furthermore, no significant difference was found between patients and HV. Interestingly, as for cell lines-derived sEV, we observed high glycosylated PD-L1 in sEV lysates derived from patients and HV (Fig. 2I). To evaluate the immunomodulatory activity of plasma-derived sEV on T cells, we developed a 7-color antibody panel for functional phenotyping of CD4^+^ and CD8^+^. Efficiency of our protocol was demonstrated by a strong expression of several early activation markers (i.e. CD25, CD69 and PD-1) after T cell activation (Fig. 2J). When plasma sEV derived from 2 patients and 2 HV were co-cultured with allogenic T cells, we observed a downward trend in activated T-cell percentages and notably for the CD25^+^CD69^+^ T cells, without effect on viability (data not shown). This reduction was observed for CD4^+^ and CD8^+^ T cells with sEV of patients but also HV (Fig. 2J), that could be explained by the high level of PD-L1 found also in HV-derived sEV.

In conclusion, we demonstrated the immunosuppressive role of sEV derived from several EBV-transformed B cells that involved cell–cell interaction and probably at least PD-L1. Furthermore, we showed that circulating sEV from DLBCL patients exhibited high CD20 and glycosylated-PD-L1 levels which may explain immunotherapy resistance. Our results suggest the benefit of using peripheral sEV in monitoring cancer progression, notably to have indirect information of the immunotherapeutic target level of parental tumor cells.

### Supplementary Information

Supplementary Material 1.

## Data Availability

Materials and methods are detailed in the supplementary information, and all data relevant to the study are included in the article or uploaded as supplementary figures.
