# Supplementary material for: Analysis of CD20 and PD-L1 levels on small extracellular vesicles (sEV) produced by DLBCL cells and EBV-transformed B cells, and potential role in T cell inhibition
Source: Exp Hematol Oncol. 2024 May 17;13:53. doi: 10.1186/s40164-024-00518-2 (PMC11100054; doi:10.1186/s40164-024-00518-2)
Supplement: Supplementary file 1 — Supplementary Material 1. [file 40164_2024_518_MOESM1_ESM.docx]

**Supplementary Material and methods**

**Cell lines**

Two GCB-DLBCL (SUDHL4 and SUDHL6 obtained from DSMZ) and three ABC-DLBCL cell lines (OCI-LY3 and OCI-LY10, obtained by Pr. Feuillard from Louis M. Staudt, National Cancer Institute, USA and U2932 from DSMZ) were cultured as previously described [9]. Five Epstein-Barr Virus (EBV)-immortalized lymphoblastoid cell lines (LCL) established in our laboratory from primary human peripheral B lymphocytes were also used and cultured as previously reported [11]. Cell lines were routinely tested to confirm the absence of mycoplasma by the MycoAlert Kit from Lonza.

**Plasma of DLBCL patients and healthy volunteers**

Blood was collected from 15 DLBCL patients at diagnosis (mean age of 68 years, range: 38-91 years) before treatment in the hematology department of Dupuytren Hospital (Limoges, France) and from 17 age-matched and sex ratio of Healthy Volunteers (HVs). Tumors were classified according to the World Health Organization classification and assigned as GCB (N=7) or non-GCB subtype (N=8) using the Hans algorithm^[[1]](#footnote-1)^ as previously described^[[2]](#footnote-2)^. CD20 positivity of biopsy samples was included in the routine IHC diagnosis practice of Dupuytren Hospital pathologists. This study was performed after approval of the Human Subjects Protection Review Board (Comité de Protection des Personnes CPP EST I Dijon, -ID-RCB : 2019-A00651-56). The trial is registered in the ClinicalTrials.gov registry (ID: 87RI18_0025 (ExoReBLy) NCT03985696). All participants signed an informed consent prior to blood draws. sEV were collected from EDTA blood samples from patients. Plasmas were obtained after two centrifugations at 2000×g for 5 min at room temperature (RT), and within 2h of collection. Plasma samples were then stored at –80°C at the CRBioLim of the Hematology Laboratory of Limoges’ CHU until use. All samples were subjected to only one freeze/thaw cycle.

**Purification and characterization of sEV containing exosomes**

DLBCL cell lines or LCLs were cultured in RPMI 1640 with 10% EV-depleted FBS by ultracentrifugation. sEV were harvested from 72h supernatants using a combination of centrifugation, ultracentrifugation (SW-28 swinging-Bucket rotor from Beckman Coulter) and filtration (0.22 μm filter) as previously reported [9]. The final pellets, that contained all sEV including exosomes, were resuspended in: Phosphate Buffered Saline (PBS) for structural and quantitative analysis with the NanoSight technology, sample buffer for ELISA, lysis buffer (Cell Signaling Technology) for western blotting analysis.

For sEV purification from plasma samples, 0.5 to 1 ml of plasma was used and diluted in PBS before submitting to a differential centrifugation and ultracentrifugation protocol (2000 g for 20 min then 16,500 g for 45 min followed by 2 ultra-centrifugations at 120,000 g for 1h20 at 4°C with rotor MLA 130 from Beckman Coulter). Alternatively, for PD-L1 analysis by ELISA, peripheral sEV were isolated from 1 ml of plasma using Total exosome isolation kit (Invitrogen) following the manufacturer’s instructions.

**Nanoparticle tracking analysis (NTA)**

Size distribution and quantitative analyses of EV from cell culture supernatants or plasma samples was performed using the NanoSight NS300 instrument (Malvern Instruments Company, Nanosight, Malvern) as previously reported [9]. Five videos of 60s were taken under controlled fluid flow with a syringe pump speed set to 100 at 25°C.

**Phenotypic characterization of sEV**

sEV preparations from DLBCL and LCLs cell cultures or plasma samples were resuspended in lysis buffer and then quantified, when possible, by measuring total protein concentration (Bio-Rad DC Protein Assay or Pierce BCA protein assay, Thermo Fisher). Expression of sEV proteins was analyzed by western blotting as previously described [9]. The following primary antibodies (Abs) were used: anti-CD81, anti-PD-L1 (H-130) from Santa Cruz Biotechnology, anti-CD63 and anti-TSG101 from Abcam, anti-ALIX and anti PD-L1 (E1L3N) from Cell Signaling Technology, anti-CD20 (Dako) and anti-βactin from Sigma. Horseradish peroxidase –conjugated secondary antibodies were from DakoCytomation. Blotted proteins were detected and quantified using the Immobilon Western Chemiluminescent HRP Substrate (Millipore) and a bioimaging system (GeneSnap, Syngene or ChemiDoc imaging system, Bio-Rad).

**Analysis of CD20 and PD-L1 surface expression on cell lines**

Cells (1x10^6^) have been incubated with anti-CD20-PE or its isotypic control (Beckman Coulter), or anti-PD-L1-PE or its isotypic control (Biolegend) for 30 min at 4°C. After washing in PBS, cells were resuspended in PBS and then analyzed by flow cytometry using FACSCalibur (Becton Dickinson), LSR Fortessa (Becton Dickinson) or Cytoflex (Beckman Coulter) flow cytometer.

**Immunofluorescence staining for confocal microscopy analysis**

Cells were fixed with 4% formaldehyde for 15 min at RT, washed with PBS and permeabilized with ice-cold 100% methanol for 10 min at -20°C. After PBS wash, non-specific binding was blocked with 1X PBS- 5% Bovine Serum Albumin (BSA)- 0.3% Triton X-100 for 60 min at RT. Cells were then incubated overnight at 4°C with primary Abs: anti-CD63 (Biolegend) and rabbit anti-PD-L1 (Cell signaling technology, clone E1L3N that recognizes endogenous levels of total PD-L1 protein.). Cells were washed with PBS followed by 633nm Alexa Fluor-goat anti-rabbit and 488nm Alexa Fluor-goat anti-mouse secondary Abs (Invitrogen) incubation for 2 h at RT. Cells were then deposited on slides using Cytospin (ThermoFisher) and mounted with VECTASHIELD mounting medium with DAPI. Pictures were taken using a confocal microscope (ZEISS LSM-800) at BISCEM technology platform, University of Limoges.

**Flow cytometry analysis of PD-L1 and CD20 expression on sEV**

sEV preparations from DLBCL and LCLs cell cultures (10 µg of total protein) were immunocaptured using magnetic Dynabeads coated with either CD63 Ab or CD81 Ab according to the manufacturer's recommendations (Invitrogen). Then bead-bound sEV complexes were stained with anti-CD20-PE (Beckman Coulter) or anti-PD-L1-PE antibodies (Biolegend) and/or costained with anti-CD63-BV421 (Biolegend) or anti-CD81-APC (Biolegend) or isotype control conjugated antibodies, followed by flow cytometry analysis using FACSCalibur, LSR Fortessa or Cytoflex flow cytometer.

**Quantification of sEV CD20 and PD-L1 levels by ELISA**

CD20 level of sEV derived from DLBCL cells (10x10^6^ cells) or plasma samples (1 ml) were quantified using CD20/MS4A1 (human) ELISA kit (BioVision) as previously reported [9]. PD-L1 level (plasma sEV) was determined using the Human PD-L1 ELISA Kit (Abcam) according to the manufacturer’s instructions.

**Analysis of LCL-derived sEV uptake by human primary T cells**

Peripheral blood mononuclear cells (PBMCs) were harvested from healthy donors by centrifugation on lymphocyte medium separation (Eurobio), and peripheral T cells were separated using negative immunoselection (Human T Cell Isolation kit; STEMCELL), following the manufacturer's instruction. The purity of recovered CD3^+^ T cells was confirmed by flow cytometry during each experiment. T cells were then stimulated or not for 24h with a cocktail of anti-CD2/anti-CD3/anti-CD28 antibodies-coated beads using T Cell Activation Expansion Kit (MACS Miltenyi Biotec), following the manufacturer's instruction. To analyse sEV uptake, sEV (5 µg total protein), labeled using the PKH67 Green Fluorescent Membrane Labeling kit (Sigma Aldrich), were incubated with stimulated and unstimulated primary T cells for 2 h. Then, the fluorescence intensity of PKH67 was analyzed by flow cytometry using FACSCalibur. Alternatively, uptake of PKH67-labeled sEV was also analyzed by confocal microscopy.

**Effect of sEV on autologous T cell viability**

Primary autologous T cells were separated from PBMCs as described above. Activated T cells (2.10^5^ cells per well in 96-well plate) were then cultured with or without LCL-derived sEV (5 µg total protein) for 24 and 48 h. Then T cells were harvested, stained with anti-CD4-PE and anti-CD8-APC (Table 1). Apoptosis was measured in CD4^+^ and CD8^+^ T cells with AnnexinV-FITC / 7-AAD staining using BD LSR Fortessa. All flow cytometry data analyses were performed using Beckman Coulter Kaluza V2.1 software.

**Immunomodulatory activity of plasma-derived sEV on primary activated T cells**

Activated or not activated primary T cells (2.10^5^ cells per well in 96-well plate) were cultured for 48 h in the presence or not of sEV (5 µg total protein) derived from plasma samples of DLBCL patients and healthy volunteers. Then cells were harvested, stained with activation T cell panel (Table S1) and analyzed by flow cytometry using Cytoflex from Beckman Coulter.

**Analysis of sEV binding capacity to therapeutic antibodies**

sEV preparations from DLBCL and LCLs cell cultures (produced by 40x10^6^ cells) were immunocaptured using magnetic Dynabeads coated with either CD63 Ab (Invitrogen, Cat N° 10606D) or CD81 Ab (Cat N° 10616D) according to the manufacturer's recommendations. Then bead-bound small EV complexes were stained with rituximab-Alexa488 or Obinutuzumab (GA101)-Alexa488 (Roche Glycart) or isotypic-Alexa488 antibody (human-IgG-A488, Dendritics). Finally, flow cytometry analysis was performed using FACSCalibur flow cytometer.

**Statistical analyses**

Significances between groups were done using a Student’s t test and one-way analysis of variances (ANOVA) with statview (Abacus Concepts). *P* < 0.05 was considered statistically significant.

**Table S1:** **Flow cytometry panel used for monitoring T cell activation.**

**Supplementary Figures**

**Fig. S1.** **LCLs-derived sEV bind therapeutic anti-CD20 antibody** **Flow cytometry analysis of the binding capacity of sEV-derived from LCLs to rituximab (RTX).** LCLs (J1209, C0404 and C1504)-derived sEV were stained with alexa-488 labeled rituximab after immunocapture. Cytograms show rituximab positive bead-bound sEV complexes in the gated population, and % of RTX^+^ complexes are indicated for each cell line.

**Fig. S2.** **Flow cytometry analysis of the binding capacity of sEV-derived from DLBCL cell lines to rituximab (RTX) and Obinutuzumab (GA101, DLBCL cell lines). (A)** Upper, comparative flow cytometry analysis of the binding of two Alexa-488 labeled anti-CD20 (rituximab and GA101, Roche Glycart) on GCB (SUDHL4/6, 4 or 6) and ABC (OCI-LY3/10 and U2932) cell lines. Cells were stained with alexa-488 labeled rituximab or GA101. Isotypic controls have been performed but are not shown to avoid cluttering the figure. Middle: sEV samples, released by 40.10^6^ cells (72h of cell culture) of **(a)** OCILY3, **(b)** SUDHL6, **(c)** SUDHL4, **(d)** OCILY10 and **(e)** U2932 cell line, were immunocaptured using magnetic beads coated with anti-CD81. Then bead-bound sEV were stained with rituximab or GA101-Alexa-488 followed by flow cytometry analysis. Example of scatterplots and fluorescence histograms are shown. The MFI of anti-CD20 positive bead-bound sEV complexes in the gated population (singlet complexes) is indicated. **(B)** Representative histograms showing MFI of beads alone or beads with only anti-CD20-Alexa48

**Figure S3. Gating strategy and flow cytometry analysis of human CD4^+^ and CD8^+^ T cell activation phenotype.** PBMC from healthy volunteers were isolated and peripheral T cells were separated using negative immunoselection. A first gate was set based on forward versus side scatter (not shown), then viable cells (7-AAD negative) were carried forward for selection of CD3^+^ cells among which we identified CD4^+^ or CD8^+^ lymphocytes. Within these 2 populations, we analyzed markers commonly related to T cell activation (i.e. CD25, CD69 and PD1 expression) in naïve **(A)** T cells or **(B)** 24h post stimulation with anti-CD3/CD2/CD28 antibodies-coated beads. Positive cell % are indicated for each marker as compared to respective isotype Ab staining.

1. Hans CP, Weisenburger DD, Greiner TC, Gascoyne RD, Delabie J, Ott G, Müller-Hermelink HK, Campo E, Braziel RM, Jaffe ES, et al. Confirmation of the molecular classification of diffuse large B-cell lymphoma by immunohistochemistry using a tissue microarray. Blood. 2004;103(1):275-282. doi:10.1182/blood-2003-05-1545. [↑](#footnote-ref-1)
2. Dubanet L, Bentayeb H, Petit B, Olivrie A, Saada S, de la Cruz-Morcillo MA, Lalloué F, Gourin MP, Bordessoule D, Faumont N, et al. Anti-apoptotic role and clinical relevance of neurotrophins in diffuse large B-cell lymphomas. Br J Cancer. 2015;113(6):934-944. doi: 10.1038/bjc.2015.274. [↑](#footnote-ref-2)
